# Phosphatidylinositol Stabilizes Fluid-Phase Liposomes Loaded with a Melphalan Lipophilic Prodrug

**DOI:** 10.3390/pharmaceutics13040473

**Published:** 2021-04-01

**Authors:** Daria Tretiakova, Irina Le-Deigen, Natalia Onishchenko, Judith Kuntsche, Elena Kudryashova, Elena Vodovozova

**Affiliations:** 1 Shemyakin–Ovchinnikov Institute of Bioorganic Chemistry, Russian Academy of Sciences, ul. Miklukho-Maklaya 16/10, 117997 Moscow, Russia; daria@lipids.ibch.ru (D.T.); natalia@lipids.ibch.ru (N.O.); 2 Department of Chemistry, Lomonosov Moscow State University, Leninskie Gory 1/3, 119991 Moscow, Russia; i.m.deygen@gmail.com (I.L.-D.); helena_koudriachova@hotmail.com (E.K.); 3 Department of Physics, Chemistry and Pharmacy, University of Southern Denmark, M Campusvej 55, 5230 Odense, Denmark; kuntsche@sdu.dk

**Keywords:** nanosized liposomes, lipophilic prodrug, melphalan, phosphatidylinositol, albumin, ATR-FTIR spectroscopy, asymmetrical flow field-flow fractionation, formulation stability

## Abstract

Previously, a liposomal formulation of a chemotherapeutic agent melphalan (Mlph) incorporated in a fluid lipid bilayer of natural phospholipids in the form of dioleoylglyceride ester (MlphDG) was developed and the antitumor effect was confirmed in mouse models. The formulation composed of egg phosphatidylcholine (ePC), soybean phosphatidylinositol (PI), and MlphDG (8:1:1, by mol) showed stability in human serum for at least 4–5 h. On the contrary, replacing PI with pegylation of the liposomes, promoted fast dissociation of the components from the bilayer. In this work, interactions of MlphDG-liposomes with the most abundant plasma protein—albumin—in function of the presence of PI in the formulation were explored using Fourier transform infrared spectroscopy. The release of MlphDG from the liposomes was studied by asymmetrical flow field-flow fractionation (AF4) using micelles formed by a polyethylene glycol conjugate with phosphatidylethanolamine to mimic the physiological lipid sink like lipoproteins. Our results show that PI actually protects the membrane of MlphDG-liposomes from the protein penetration, presumably due to pairing between the positively charged MlphDG and negatively charged PI, which compensates for the heterogeneity of the lipid bilayer. The AF4 technique also evidences high stability of the formulation as a drug carrier.

## 1. Introduction

Liposomes lead the way among drug delivery systems designed for administration into the bloodstream due to their exceptional bio- and hemocompatibility [1,2]. However, in circulation, liposomes, similar to other drug delivery systems, are instantly covered with a complex layer of proteins and lipoproteins [3,4]. Plasma proteins adsorb on surface of liposomes through either specific or non-specific interactions. The liposome thus gains new identity exposing the adsorbed proteins on the surface [5]. The adsorbed proteins mediate the liposome interactions with other proteins, including cell receptors [6]. This protein corona usually contains opsonins, which promote phagocytosis by leucocytes. Premature withdrawal of nanoparticulate carriers from circulation by myeloid cells represents the main barrier for drug delivery to target tissues [7]. Additionally, in the case of liposomes, contact with plasma proteins can lead to disturbance and disintegration of the lipid bilayer followed by the leakage of the cargo. The most recognized approach to circumvent opsonization is coating of the nanoparticles, including liposomes, with polyethylene glycol (PEG) [2,8]. The technology continues to be upgraded and a process using supercritical fluids has recently been proposed for physical adsorption of PEG on liposome surface [9]. Pegylation, however, does not prevent non-specific binding of proteins, which often results in complement activation-related infusion reactions of varying severity [10,11] and other anti-PEG immunity-associated issues [12].

**Figure 1:**
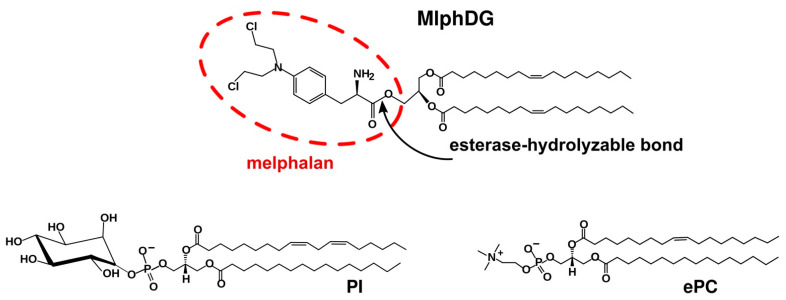
Structure of melphalan lipophilic prodrug (MlphDG) and representative structures of soybean phosphatidylinositol (PI) and egg phosphatidylcholine (ePC) used for liposome preparation.

Earlier, a liposomal formulation of melphalan incorporated in a fluid liposome bilayer composed of natural phospholipids in the form of a lipophilic prodrug, i.e., the dioleoylglyceride ester conjugate of melphalan (MlphDG, Figure 1), has been developed [13]. Melphalan, 4-[bis(2-chloroethyl)amino]-L-phenylalanine, also known as L-phenylalanine mustard, is a cell-cycle non-specific alkylating cytotoxic drug. Owing to the broad spectrum of antitumor activity, it has been used in chemotherapy of malignancies for over 50 years [14,15]. Poor solubility and low stability at physiological pH, as well as rapid removal from circulation, require administration of high doses of melphalan, which causes multiple side effects. Encapsulation of melphalan in a nanocarrier can improve its pharmacological characteristics. Several studies on successful incorporation of melphalan as an active pharmaceutical ingredient in polymeric [16,17] or solid lipid PEG-coated nanoparticles [18,19] have been reported recently. As for nanosized liposomes, passive encapsulation of even highly water-soluble drugs is inefficient. Remote loading techniques provide for therapeutically relevant concentrations of water-soluble drugs in liposome inner volume, but they can only be applied to weak amphipathic bases or acids [20], which melphalan does not belong to. Harnessing lipophilic prodrugs simplifies fabrication of lipid-based nanomedicines and provides for feasible loading capacity thereof [21,22]. Besides, alkylating radicals inserted into lipidic environment are protected from hydrolysis, as reported for the chlorambucil lipophilic prodrug in a liposomal formulation [23].

Lipid bilayer of MlphDG-liposomes is formed by egg phosphatidylcholine (ePC) and phosphatidylinositol (PI) (Figure 1). PI is added to the formulation because of its ability to prolong circulation half-life of nanosized liposomes [24,25]. In early in vitro tests of ePC–PI liposomes loaded with 10 mol% MlphDG in the bilayer the formulation showed good hemocompatibility and did not activate complement system [26]. MlphDG-liposomes were more effective than free melphalan in inhibiting tumor growth in a Lewis lung carcinoma model [27] and in a transplanted WNT-1 tumor from genetically engineered breast cancer model [28] in mice; in rats, the liposomes were less toxic and better tolerated [28].

To further enhance the performance of the fluid-phase liposomal formulation of MlphDG, the effect of various stabilizing components introduced in the bilayer was explored [29]. According to the calcein leakage assay, the presence of PI (10 mol%) ensured stabilization of the liposomal formulation in serum for at least 4–5 h, in contrast to pegylation (inclusion of a PEG–lipid conjugate), which promoted dissociation of the components from the bilayer. (As expected [30], the PEG–lipid conjugate only protected MlphDG-liposomes composed of distearoylphosphatidylcholine and cholesterol forming the so-called liquid ordered (LO) phase [29]. However, preparation of the liposomes with bilayer in LO phase requires heating, which is unwanted because it promotes degradation of reactive alkylating groups of melphalan.)

Serum albumin is the most abundant protein of blood plasma. It is a helical protein with highly flexible structure. Primary natural ligands of albumin are fatty acids. However, it binds a wide range of cationic and anionic hydrophobic compounds utilizing both hydrophobic and electrostatic interactions. Depending on the phospholipid nature, albumin can either be stabilized (as in the presence of dioleoylphosphatidylethanolamine [31]) or partially denatured (as when interacted with DMPC [32] or DOTAP [31]) by the interaction. As for the lipid membranes, serum albumin has been shown to bind to (DMPC [32] and DPPC [33] liposomes) and even penetrate (DPPC, DMPC, POPC, or DOPC [34]) phospholipid bilayers leading to increased mobility of the charged head groups and disturbance of acyl chain packing as well.

Here, attenuated total reflection (ATR) Fourier transform infrared (FTIR) spectroscopy (ATR-FTIR spectroscopy) was used to track the interactions of MlphDG-loaded ePC–PI liposomes with serum albumin and the changes they induce in the structure of albumin on the one hand and phospholipid bilayer on the other. ATR-FTIR spectroscopy is a surface-sensitive version of infrared spectroscopy that is particularly informative in studying protein–membrane interactions at sub-molecular level [35]. We also employed asymmetrical flow field-flow fractionation (AF4) [36] to estimate the prodrug leakage from the bilayer. This technique has been proven a very useful analytical tool to estimate drug release properties especially for highly hydrophobic, water-insoluble drugs where the mechanism of drug release is mainly due to collision [37].

## 2. Materials and Methods

### 2.1. Chemicals

Egg phosphatidylcholine (ePC; USP grade, Lipoid E PC S) and DSPE-PEG2000 were obtained from Lipoid GmbH (Heidelberg, Germany); raw soybean phosphatidylinositol (PI) was a kind gift from Lipoid, it was further purified by column chromatography on silica gel and characterized by 1H-NMR spectroscopy as an individual phospholipid; dioleoylglycerol ester melphalan conjugate (MlphDG) [28] and bis-cyclohexyl-BODIPY-labeled phosphatidylcholine (BCHB-PC) [38] were synthesized as previously reported. Bovine serum albumin (BSA) was purchased from PanEco (Moscow, Russia).

### 2.2. Preparation of Liposomes

Liposomes (large unilamellar vesicles) were prepared as described earlier [13,26,27]. Briefly, lipid films were obtained by co-evaporation of aliquots of stock solutions of lipids and MlphDG in chloroform–methanol (2:1) in round-bottom flask on a rotary evaporator, with subsequent drying for 45 min at 5 Pa. The resulting compositions were ePC; ePC–PI, 9:1; ePC–MlphDG, 9:1; ePC–MlphDG–PI, 8:1:1 (by mol). If not stated otherwise, the lipid films were hydrated in phosphate buffered saline (PBS, pH 7.4), subjected to seven cycles of freezing/thawing (liquid nitrogen/+40 °C), and extruded 20 times through Whatman Nuclepore track-etched polycarbonate membranes (Cytiva, Marlborough, MA, USA) with a pore size of 100 nm on a mini-extruder (Avanti, Alabaster, AL, USA). Phospholipid concentrations in liposome dispersions were measured by the colorimetric assay [39]. Prodrug concentrations were controlled by UV spectrophotometry after liposome disruption with ethanol (λ_max_ MlphDG 260 nm, ε 16,100 M^−1^ cm^−1^). For Förster resonance energy transfer (FRET) experiments, 1 mol% bis-cyclohexyl-BODIPY-labeled phosphatidylcholine (BCHB-PC) was added at the stage of lipid film formation.

Liposome dispersions were stored at 4 °C and used for experiments within 7 days.

### 2.3. Hydrodynamic Diameter and Zeta Potential Measurements

Particle size was measured by dynamic light scattering (DLS) with Zetasizer Nano ZS (Malvern Panalytical Ltd., Malvern, UK) or DelsaMAX Pro instrument (Beckman Coulter, Brea, CA, USA) with green laser (532 nm, 90°). Aliquots of extruded liposomes were diluted in buffer to about 0.05 mg/mL and aliquots of micelles to about ~0.1 mg/mL. Samples were measured 10 times over 30 s at 20 °C. Average particle size and width of the particle size distribution were determined by cumulant method. For reliable measurements of zeta potential, liposome samples with diameters of around 200 nm were prepared in 10 mM KCl, 1 mM K_2_HPO_4_, 1 mM KH_2_PO_4_, pH 7.0 buffer (extruded 20 times through 200 nm polycarbonate membrane filters). Zeta potential values were obtained using ZetaPALS analyzer (Brookhaven Instruments Corp., Holtsville, NY, USA; provided by the Core Facility of the Institute of Gene Biology, Russian Academy of Sciences, Moscow, Russia). Samples of the liposomes (1.5 mL, 1 mg/mL total lipids) were equilibrated for 1 min in cuvettes before 10 runs of 25 cycles per sample were performed at 25 °C. Zeta potential values were calculated using Smoluchowski approximation.

### 2.4. ATR-FTIR Spectroscopy

ATR-FTIR spectra were recorded using a Bruker Tensor 27 spectrometer equipped with a liquid nitrogen-cooled MCT (mercury cadmium telluride) detector. Samples were placed in a BioATR-II temperature-controlled cell with ZnSe ATR (attenuated total reflection) element (Bruker, Germany). The ATR-FTIR spectrometer was purged with a constant flow of dry air. ATR-FTIR spectra were registered from 900 to 3000 cm^−1^. For each spectrum, 80 scans were accumulated at 20 kHz scanning speed and averaged. All spectra were registered in PBS, pH 7.4, at 37 °C, lipid concentration was 12 mg/mL, volume applied to ZnSe element was 40 μL. To study changes in protein and lipids spectral characteristics upon incubations, spectra of pure BSA, pure liposomes, and BSA–liposome mixture were acquired. Albumin spectra were obtained for 5 μL BSA aliquot (50 mg/mL) diluted with 35 μL PBS (pH 7.4). Intact liposome spectra were obtained for 10 μL liposome sample aliquot (50 mM) diluted with 30 μL PBS. To study the effect of albumin on the bilayer, 5 μL BSA and 10 μL liposomes were mixed, sample volume was adjusted to 40 μL with PBS, and afterwards the mixture was incubated on the ZnSe element. Spectra of liposome–protein complexes were recorded after 3, 5, 10, 15, and 20 min of incubation. The experiment was repeated twice. Spectral data were processed using the Opus 7.5 (Bruker, Germany) software system, which includes linear blank subtraction, straight-line baseline correction, and atmosphere compensation. If necessary, Savitsky–Golay smoothing was used to remove white noise and wide peaks were subjected to deconvolution procedure. Peaks were identified by standard Bruker peak picking procedure.

### 2.5. Fluorescence Measurements

Fluorescein isothiocyanate (FITC)–BSA conjugate was prepared as described in [40]. FITC (Fluka, Buchs, Switzerland) was dissolved in DMSO to a final concentration of 2.6 mg/mL. BSA was dissolved in carbonate–bicarbonate buffer (pH 9.65, Na_2_CO_3_·10H_2_O, 8.586 g/L; NaHCO_3_, 5.88 g/L) to a final concentration of 2 mg/mL. An aliquot of 1 mL BSA was placed in a dark glass vial and 40 μL FITC aliquot was added slowly drop-wise under stirring, pH range was 9–10. The mixture was stirred for 16.5 h in the dark at 12 °C. Then NH_4_Cl was added to a final concentration of 50 mM to stop conjugation reaction. After 2.5 h, the reaction mixture was applied onto a Sephadex G-25 column (V = 20 mL) and eluted with PBS (pH 7.4). Three fractions with maximum intensity for BSA at λ 276 nm were pooled together. Protein and FITC concentrations were determined by UV/Vis spectrometry (FITC, λ_max_ 496 nm; ε 73,000 M^−1^ cm^−1^). Dye-to-protein ratio was determined as in [40].

For fluorescence measurements, FITC–BSA concentration in the samples was kept below 6.85 × 10^−7^ M (corresponding to the absorbance <0.05 a.u. to avoid the inner filter effect). Aliquots of liposomes with 1% BCHB-PC probe were added to the solution of FITC–BSA conjugate in PBS to get different BSA per liposome ratios. Resulting mixtures were incubated at 37 °C for 20 min in the cuvette of the F-4000 fluorescence spectrophotometer (Hitachi, Tokyo, Japan). A decrease in the FITC emission signal (λ_ex_ 470 nm, λ_em_ 523 nm) after liposome addition due to resonance energy transfer to BCHB-PC (λ 536 nm, ε 68,000 M^−1^ cm^−1^) upon protein adsorption was monitored.

### 2.6. Asymmetrical Flow Field-Flow Fractionation (AF4)

Liposomes were analyzed by an Eclipse 3+ separation system (Wyatt Technology, Dernbach, Germany) equipped with an isocratic pump, degasser, and autosampler with temperature and stirring mode controls (Agilent 1200 seroes, Agilent Technology, Dernbach, Germany). The separation channel (length 26 cm, largest width 21 mm, height 350 µm) was equipped with a regenerated cellulose membrane (MWCO 10 kDa, Wyatt Technology, Dernbach, Germany) and connected to a multi-angle light scattering detector (MALS, DAWN-Heleos II, Wyatt Technology, Dernbach, Germany), a refractive index detector (Optilab reX, Wyatt Technology, Dernbach, Germany), and a variable wavelength detector (VWD, Agilent Technology, Germany) set to 260 nm for MlphDG prodrug quantification. Phosphate buffer (10 mM KCl, 1 mM K_2_HPO_4_, 1 mM KH_2_PO_4_, pH 7.0) preserved with 0.02% NaN_3_ was used as carrier liquid and for sample dilution.

#### 2.6.1. Size Determination

An aliquot of 30 µL of diluted liposomal dispersions (15 μg injected lipid mass) was injected at 0.2 mL/min into the AF4 channel in the focus mode (focus flow 2 mL/min) and eluted at constant detector flow of 1 mL/min and the following cross flow conditions: decreasing cross flow from 2 mL/min to 0.5 mL/min over 5 min and from 0.5 mL/min to 0 mL/min over 30 min followed by elution without applied cross flow. Liposome size and size distributions were calculated by the ASTRA software (version 4.9) (Wyatt Technology, Santa Barbara, CA, USA) using hollow sphere model [41]. The size results are presented as mean diameter and D10, D50, and D90 representing the diameters at 10, 50 (median), and 90% of the cumulative mass distribution.

#### 2.6.2. Batch Analysis (Determination of Extinction Coefficient)

Aliquots of 20, 40, 60, and 80 µL diluted liposomes (lipid concentration 0.5 mg/mL) were injected into the MALS and VWD detector with a flow rate of 1 mL/min and blank liposomes (ePC–PI) with similar size were used to correct for scattering effects [42,43]. To establish a calibration curve, peak integrals of the VWD detector were corrected for scattering effects (signals from blank liposomes) and plotted against the injected MlphDG masses. The resulting extinction coefficient (0.01881 mL mg^−1^ cm^−1^) was used to quantify MlphDG in the release experiments. All experiments were done in triplicate.

#### 2.6.3. MlphDG Release

The ePC–PI, 9:1, and ePC–MlphDG–PI, 8:1:1 (*n*/*n* ratios), liposome suspensions (6.5 mg/mL lipid in phosphate buffer, 10 mM KCl, 1 mM K_2_HPO_4_, 1 mM KH_2_PO_4_, pH 7.0) were prepared as described in Section 2.2, and submitted to AF4 release experiments. DSPE-mPEG micelles were used as an acceptor and prepared by dissolving 20 mg DSPE-PEG 2000 in 2 mL phosphate buffer and heating to 70 °C for 30 min.

The ePC–MlphDG–PI liposomes were mixed with the DSPE-mPEG micelles at a 1:1 lipid mass ratio and a total lipid concentration of 1 mg/mL. The mixture was incubated at 37 °C under magnetic stirring and 30-μL samples were submitted to AF4 every hour over a period of 24 h. As controls, the same amounts of blank liposomes (0.5 mg/mL). Blank DSPE-mPEG micelles (0.5 mg/mL), and a mixture of both were analyzed. The controls were injected in triplicate to determine the impact of light scattering on the UV signals. The separation conditions are summarized in Table 1.

## 3. Results and Discussion

### 3.1. Characteristics of the Liposomes

Measured size and zeta-potential values of the liposomes are presented in Table 2.

Zeta-potential values of the membranes are consistent with the charges of the components: the inclusion of MlphDG in practically neutral ePC liposomes composed of zwitter-ionic phosphatidylcholine molecules resulted in positively charged ePC–MlphDG liposomes (+18 mV), for the primary amino group of melphalan moiety is protonated at pH 7.4 (PBS). Inclusion of 10 mol% PI in a neutral bilayer, on the contrary, produced negatively charged ePC–PI liposomes (−45 mV). The negative charge along with the steric hindrances caused by highly hydrated and bulky inositol moieties are assumed to be the cause of reduced uptake of PI-containing liposomes by cells of reticuloendothelial system [44]. As expected further, inclusion in the bilayer of MlphDG in the amount equimolar to that of PI led to partial neutralization of the negative charge and yielded the potential of −25 mV for the ePC–MlphDG–PI liposomes.

First, the interaction between liposomes and albumin was examined using Förster resonance energy transfer (FRET) using BSA–FITC conjugate as a donor and BCHB-PC probe in liposome bilayer as an acceptor molecule [45]. Provided albumin adsorbed on a liposome surface, FITC signal should decrease due to the energy transfer to BCHB-PC proportionally to the amount of the protein adsorbed, depth of its penetration in the bilayer, and/or changes of mutual arrangement of the fluorophores. FRET was registered for all liposome formulations at different protein–liposome ratios (Supplementary Materials, Figure S2), however, there were no quantitative differences in the magnitude of FRET nor did it depend on the incubation time.

### 3.2. ATR-FTIR Spectroscopy

Interactions of liposomes with albumin were evaluated from the point of changes in the structures of the lipid bilayers and the protein. Changes in the lipid packing density and physical state (e.g., gel or liquid-crystalline) can be detected using characteristic frequency (wavenumber) bands of absorption of lipid methylene, carbonyl, and phosphate groups. High sensitivity of ATR-FTIR spectroscopy in physicochemical studies of liposome complexes with small molecule drugs [46,47] and PEG–chitosan complex [48] has been demonstrated previously.

**Figure 2:**
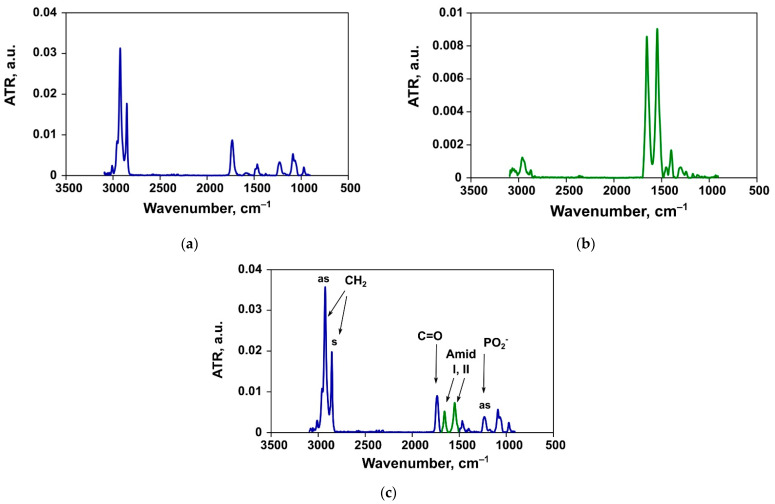
An example of the ATR-FTIR spectrum of ePC liposomes (**a**), bovine serum albumin (BSA) (**b**), and mixture thereof (**c**).

Spectra of the liposomes and albumin registered separately in PBS at 37 °C are presented in Figure 2a,b respectively. Figure 2c demonstrates an exemplar spectrum of a liposome dispersion upon addition of albumin during a 20-min incubation at 37 °C.

#### 3.2.1. Changes in the Structure of Lipid Bilayer

In the polar region of membrane, frequencies of stretching bands of lipid carbonyl (1700–1750 cm^−1^) and phosphate (1220–1260 cm^−1^) groups respond to changes in the microenvironment of bilayer, such as breaking the bonds with water molecules and formation of bonds with incoming proteins or other ligands [49,50,51]. Asymmetric and symmetric (~2920 cm^−1^ and ~2850 cm^−1^, respectively) stretching vibrations of CH_2_ groups are sensitive to changes in the hydrophobic region of membrane and therefore reflect the bilayer package [52]. Analytically significant values are observed for methylene and carbonyl groups. Interpretation of the bands of phosphate groups is hampered by the complicated analysis of the peak after deconvolution, however, may provide valuable insights.

##### Asymmetric Stretching Vibrations of Phosphate Groups

Phosphate group bands in the liposomes formed a wide multicomponent peak (1220–1270 cm^−1^) with the maximum at around 1230 cm^−1^. After addition of BSA it shifted towards higher frequencies (Table 3) and changed its shape (Supplementary Materials, Figure S3). The shift of the PO_2_^−^ peak maximum could be interpreted as breaking of bonds between the lipids and water molecules upon protein adsorption and formation of new bonds with the protein.

Most prominent changes in the PO_2_^−^ peak position was observed for the ePC–MlphDG sample, indicating strong (electrostatic) interactions of albumin with the positively charged surface of liposomes. Uncharged ePC liposomes showed insignificant if any intervention of the protein in the polar region of the bilayer. In the case of negatively charged liposomes comprising PI, albumin formed a network of bonds with the phosphate groups presumably due to rapprochement in the course of hydrogen bond formation with inositol moieties. (Hypothetically, the formation of bridges due to traces of divalent cations in the medium could also contribute to the bonding between negatively charged amino acid residues and the phosphate groups). Notably, inclusion of MlphDG in the PI-containing bilayer reduced the effect of BSA on the bilayer (the PO_2_^−^ peak maximum shifted by 2.5 cm^−1^ in contrast to 3.4 cm^−1^ for the ePC–PI liposomes, Table 3).

##### Ester Carbonyl Stretching Bands

**Figure 3:**
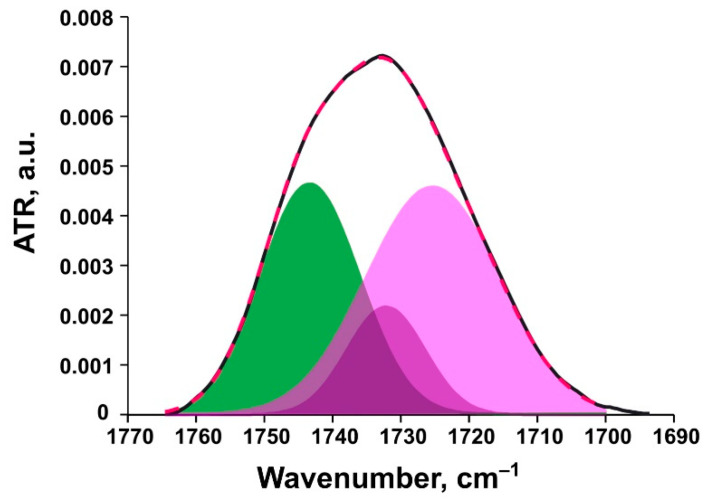
Experimental ester carbonyl peak for ePC liposomes (black line) and deconvolution components (green, free carbonyl groups; purple, highly hydrated carbonyl groups) with resulting fitting curve shown as a red dashed line.

Bands of carbonyl groups (1720–1750 cm^−1^) are multicomponent: high-frequency components correspond to low hydrated groups and low frequency bands correspond to highly hydrated groups. A decrease in hydration degree is usually caused by formation of electrostatic bonds with charged ligands. Ester carbonyl stretching band is a superposition of at least two main components: free C=O group centered at ~1741 cm^−1^ and hydrogen-bound C=O at ~1727 cm^−1^ [49]. As reported by [53], in fully hydrated liposomes, the carbonyl absorption band is located at 1725 cm^−1^, while in completely dehydrated liposomes (dry film), it shifts toward higher wavenumbers and equals 1745 cm^−1^. In order to analyze the signals of carbonyl groups, the spectrum in the region 1700–1760 cm^−1^ (Figure 3 and Supplementary Materials, Figure S4) was deconvoluted and the percentage of hydrogen-bound carbonyl groups was calculated.

**Figure 4:**
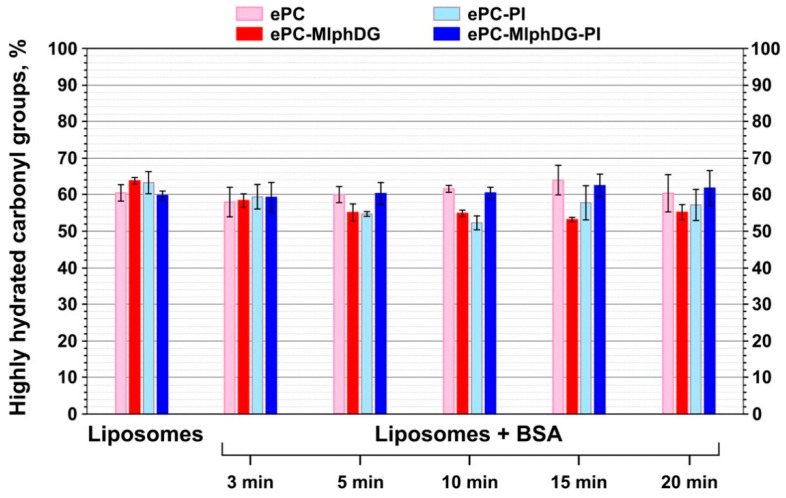
Changes in the percent of highly hydrated carbonyl groups for liposomes of different compositions prior to and upon their incubation with BSA. Mean values ± SE for two independent experiments are presented.

All carbonyl peak subcomponents with frequencies lower than or equal to 1733 cm^−1^ were referred to the high hydration degree groups. Sum of their peak areas represents the percent of the so-called highly hydrated carbonyl groups in a liposome sample. Ester carbonyls in liposomal formulations had approximately the same amount of bonds with water prior to and after the addition of BSA (Figure 4).

During incubation with BSA, changes in the peak subcomponents of the ester carbonyls and the percentage of hydrogen-bound groups for the ePC or ePC–MlphDG–PI liposomes seem to be not prominent. The addition of albumin to ePC–PI or ePC–MlphDG liposomes appears to break some of the lipid–water bonds, as there is a downward trend in the percent of highly hydrated carbonyls (Figure 4). In the case of the ePC–MlphDG liposomes, the decrease is somewhat more pronounced. This indicates that the protein binding to the liposomes promotes partial displacement of water molecules from glyceride carbonyls. Probably, the introduction of PI or MlphDG alone in the ePC liposomes somewhat disrupts the regular structure of the bilayer surface composed of individual phosphocholine head groups, leading to higher exposure of the glyceride carbonyls for binding with water compared to pure ePC. The defects in the surface of membrane allow the protein to dive in the subpolar region of the lipid bilayer and dehydrate a part of carbonyls. When both PI and MlphDG are present in the bilayer in equal amounts, interactions between the relatively small and positively charged melphalan moieties of the prodrug and large negatively charged *mio*-inositol head groups of PI probably shield the carbonyls from binding with water or protein hydrogens.

##### Methylene Stretching Vibrations

**Figure 5:**
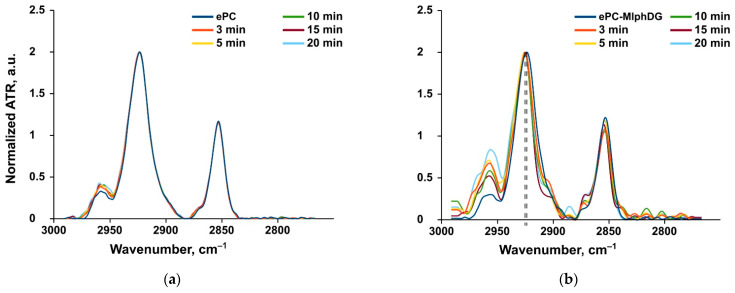
Examples of FTIR spectra. Methylene peaks for the ePC (**a**) and ePC–MlphDG (**b**) liposomes alone and upon their incubation with BSA. Dashed lines show the peak shift for the ePC–MlphDG liposomes upon the addition of albumin.

Methylene stretching vibrations are generally the strongest bands in the spectra of lipids. These frequencies are sensitive to trans/gauche orientation changes in the lipid acyl chains. According to [51], CH_2_ symmetric stretching band may be preferable to monitor because it is freer from the overlap by other vibrational modes. The frequencies of methylene stretching vibrations are known to increase during gel-to-liquid crystalline phase transitions of lipids. In particular, the asymmetric CH_2_ oscillation band shifts from ~2916 to ~2924 cm^−1^ upon phase transition and increase of chain disorder due to the increase of gauche conformation [51]. There is an inverse relationship between the lipid packing densities and CH_2_ stretching frequencies [52]. For the monolayers of 1-palmitoyl-2-oleoyl-sn-glycerophosphocholine, this vibrational mode was detected at 2922.8 cm^−1^, which confirmed the fluid-like state of the lipid [52]. As expected, the frequency of asymmetric CH_2_ stretching vibrations of the ePC liposomes also corresponded to the liquid disordered phase of lipid bilayer (2923.6 cm^−1^, Table 4). Incubation of the ePC liposomes with albumin did not influence any methylene vibrations (Table 4, Figure 5a). Thus, the protein adsorption did not disturb the uniform liposome bilayer.

The introduction of MlphDG prodrug molecules into the ePC bilayer affected the response of methylene stretching vibrations to the addition of albumin. During incubation of the ePC–MlphDG liposomes with albumin both asymmetric and symmetric CH_2_ peak positions shifted towards higher frequencies range (Table 4, Figure 5b). This increase in the bilayer disorder most likely indicates the insertion of the protein in the bilayer as a result of interactions with the positively charged prodrug molecule. The ability of albumin to penetrate liposome bilayer has been stated in other works [32,34]. According to [32], penetration may also cause conformational changes in the protein.

Incorporation of PI into the ePC bilayer practically did not influence the bilayer susceptibility to protein penetration (Table 4, Supplementary Materials, Figure S4). Although MlphDG introduced some disturbances in packing of the lipid bilayer, in the ePC–MlphDG–PI formulation bearing the prodrug in equimolar amount with PI (10% by mol), PI compensated for the prodrug effects: incubation of these liposomes with BSA did not affect the mobility of carbon chains at all, which means that the protein did not penetrate into the membrane (Table 4, Supplementary Materials, Figure S5).

#### 3.2.2. Changes in the Structure of the Protein

A polypeptide chain generates several infrared-active amide vibrational modes, among which the Amide I is one of the most sensitive to the structural dynamics of proteins [35]. Amide I mode (1600–1700 cm^−1^) is produced by the set of vibrations of chemical bonds comprising the amide group. These “oscillators are coupled to each other via covalent bonds, H bonding, and through space, all of which are distinct for different secondary structures. Consequently, different secondary structures of polypeptides produce amide I bands at distinct frequencies” [35]. Changes in the secondary structure of the protein when it interacts with the membrane can be detected as a redistribution of the signal intensities of different structural elements, such as α-helix/β-sheet/β-turn/random coil, in the Amide I mode (in our case, 1628–1685 cm^−1^). The appearance of protein aggregates in the solution corresponds to the absorption peaks of the antiparallel β-sheet in the frequency regions of approximately 1624 and 1696 cm^−1^ [54]. These individual signals of structural elements are not distinctive in spectra, they are obtained after second derivative deconvolution of the Amide I mode. In our work, a correlation between secondary structure and its subcomponent peak positions was made mainly basing on the papers by [54,55,56].

**Figure 6:**
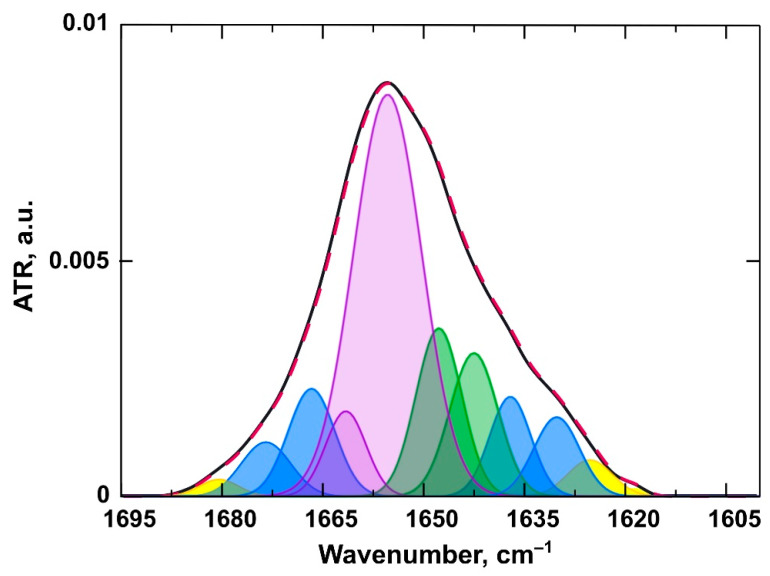
An example of the amide I band of a BSA spectrum (black line) and deconvolution components (purple, α-helix; green, random coil; blue, β-turns and sheets; yellow line, aggregates) with resulting fitting curve shown as a red dashed line.

Deconvolution of the spectrum in the region 1600–1700 cm^−1^ (Figure 6, Supplementary Materials, Figure S6) was followed by calculations of the percent of structural elements, such as α-helix/β-sheet/β-turn/random coil, as sum of relevant peak areas. The procedure of the amide I mode second derivative deconvolution yielded about 10 different subcomponents and the main ones were assigned to specific structural element in accordance with Table 5.

Thus, from pure serum albumin spectra, initial values of protein secondary structure elements contribution (in %) to the structure were calculated to identify any alterations during incubation with liposomes. Pure BSA was mainly a α-helical protein (60.0%), with 21.6% of random coil and 18.4% of β-sheet and β-turn combined (Figure 6). These average values are quite similar to the X-ray data reported in [57]. Percentage of BSA aggregates did not exceed 10% in all experiments. As the number of protein molecules per liposome was about 600, which is barely enough to create a single-layer protein corona, no additional information was obtained from the aggregate signals.

**Figure 7:**
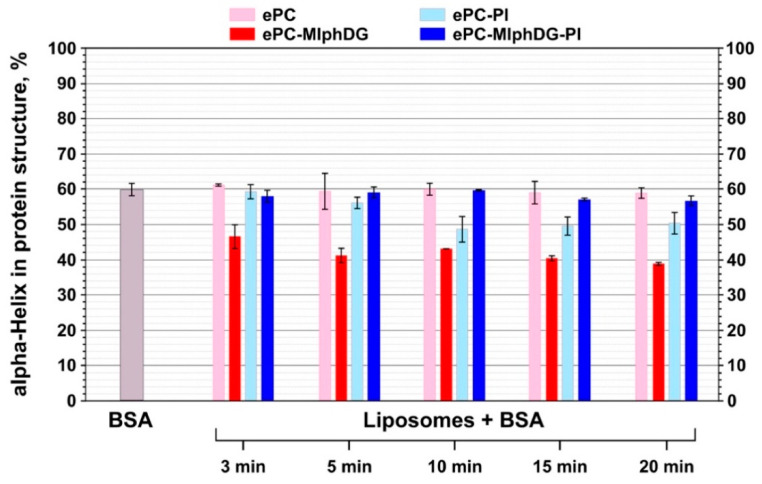
Changes in the percent of α-helices in the structure of BSA during interaction with liposomes of different compositions. Mean values ± SE of two independent experiments are presented.

The data acquired from the analysis of amide I transformations during interaction of BSA with liposomes corroborate the trends revealed by the analysis of phosphate group, ester carbonyl, and methylene stretching vibrations in liposomes. Albumin adsorption on the ePC liposomes did not cause any alterations in its secondary structure (Figure 7, pale pink bars). Probably, this is due to the neutrally charged and uniform lipid bilayer. Upon interaction with the negatively charged ePC–MlphDG–PI formulation, there was a slight decrease in the protein helicity to about 57% (Figure 7, navy blue bars). The ePC–PI and ePC–MlphDG liposomes caused more pronounced changes: the decrease of α-helix in the protein structure down to 50% and 40%, respectively. Proportion of the α-helical structures decreased along with the increase in β-structural elements and random coils. The effect of the ePC–MlphDG liposomes on the structure of the negatively charged protein (zeta potential of BSA is −35 mV) could be at least partially explained by the positive surface charge introduced by melphalan moiety. The molecules of protein on the surface of liposomes can rearrange to find the most advantageous conformation. Keeping in mind that it was the ePC–MlphDG bilayer which was penetrated by albumin, one can suppose that this new secondary structure favors the protein movement towards hydrophobic region of the bilayer. Similar decrease in α-helicity in albumin upon adsorption was observed for positively charged golden nanoparticles by Fleischer et al. [58]. Interactions of BSA with negatively charged ePC–PI liposomes also caused structural changes in the protein molecule (Figure 7, pale blue bars), though not that pronounced as in the case of ePC–MlphDG sample, which is consistent with the data on the impact of the BSA–liposome interaction on stretching vibrations of phosphate groups (Table 3) and ester carbonyls (Figure 4). The compensatory effect on packing of the bilayer (including its surface) caused by concurrent presence of phosphatidylinositol and melphalan prodrug is confirmed by minimal influence of the ePC–MlphDG–PI liposomes on the structure of adsorbed protein.

According to Fleischer et al. [58], conformationally altered albumin adsorbed on a nanoparticle may switch cellular interaction routes from its native receptor to scavenger receptor. Particularly, loss of 20% of α-helicity of BSA on the surface of positively charged polystyrene nanoparticles drove them to bind to scavenger receptors. Moreover, in early works, Schnitzer et al. suggested specific receptors that recognize conformational changes in albumin [59]. As the ePC–MlphDG liposomes caused similar changes in the structure of albumin, these data favor the use of negatively charged stabilizing components with the prodrug molecules.

### 3.3. Asymmetrical Flow-Field-Flow Fractionation (AF4)

Thus, the ePC–MlphDG–PI liposomes were proven to be practically insensitive to BSA protein corona formation and were physically stable at least for 4 h in human serum in our previous experiments [29]. In this study, AF4 was used to evaluate the potential release of the prodrug using DSPE-mPEG micelles as an acceptor phase. DPPE-mPEG micelles have been chosen due to their high stability and small size [60], thus facilitating baseline separation from the liposome fraction in AF4. The separation system was connected to multi-angle light scattering (MALS) and absorbance detection allowing monitoring of changes in size of the colloids as well as MlphDG amount in both colloidal fractions [42]. Due to the positive charge of the ePC–MlphDG liposomes (+18 mV), they could not be accurately analyzed as they had a tendency to stick on the membrane, which resulted in poor recovery. Therefore, only negatively charged ePC–MlphDG–PI liposomes were analyzed and plain ePC–PI liposomes were used as control.

Prior to the release study, preliminary AF4 experiments were carried out to characterize the liposomes and micelles to ensure adequate separation of both fractions, as well as prodrug quantification in the fractions.

**Figure 8:**
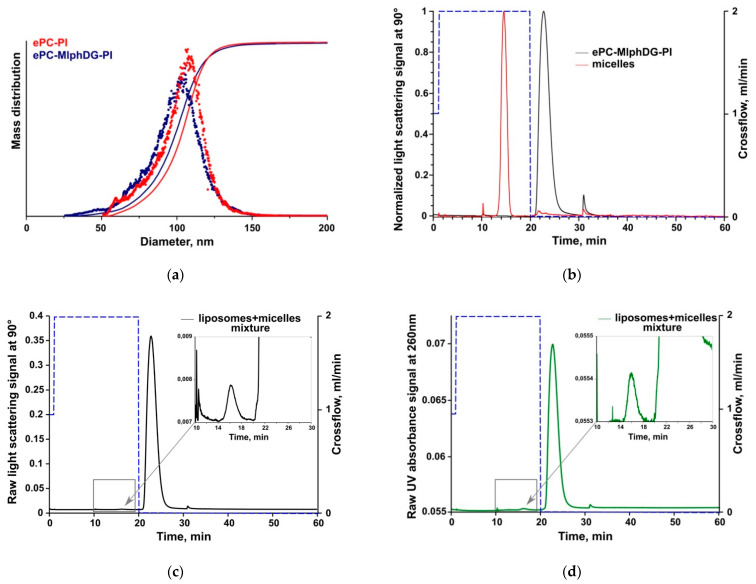
AF4/MALS liposome size distribution (**a**). AF4/MALS elution profile of micelles and ePC–MlphDG–PI liposomes injected separately (**b**) and in mixture ((**c**,**d**) respectively) where the dotted lines show the crossflow conditions. In (**b**,**c**), the light scattering signals at 90° are shown, in (**d**), the variable wavelength detector (VWD) signal at 260 nm. The first 10 min of analysis is focusing and sample injection.

Results of DLS and AF4/MALS size measurements are summarized in Table 6. Both methods produced similar data with slightly larger sizes determined by AF4/MALS. The variations were, however, less than 10 nm. Due to their small size (isotropic scattering), the size of DSPE-mPEG micelles could not be analyzed by AF4/MALS. Liposome size distribution acquired by AF4/MALS is presented in Figure 8a.

First, micelles and liposomes were analyzed separately by AF4/MALS to determine their elution times (Figure 8). The micelles were eluted at the high initial constant crossflow, while liposomes did not elute under these conditions (Figure 8b). The crossflow was kept for 10 min and turned off afterwards to elute the liposomes and maintain baseline separation of both fractions. Similar elution times were observed when liposomes and micelles were mixed and both fractions were baseline-separated (Figure 8c,d), which is an important prerequisite for the release experiments.

**Figure 9:**
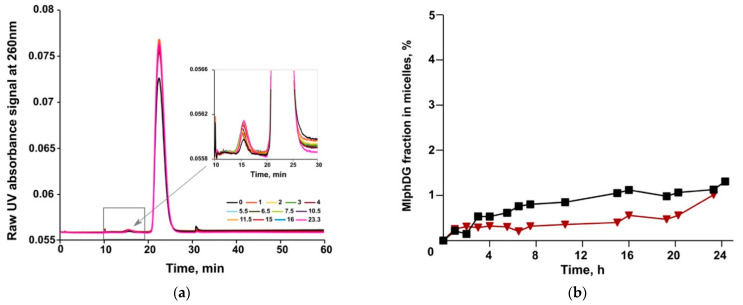
AF4/VWD elution profiles of MlphDG transfer from the ePC–MlphDG–PI liposomes to micelles taken at different time of incubation (**a**) and the amount of MlphDG (in percent to the amount injected in the liposomal formulation) transferred from liposomes to micelles (**b**).

In the drug release study, MlphDG-containing liposomes were mixed with the DSPE-mPEG micelles in a 1:1 mass ratio and samples were being submitted to AF4 every hour over a total incubation time of 24 h. Representative elution profiles (VWD signals) are shown in Figure 9. There was a slight increase in intensity (VWD signal) in the micelle fraction together with a corresponding decrease in intensity in the liposome fraction (Figure 9a) indicating only a minor release of the prodrug from the liposomes. The calculated amounts of released MlphDG (e.g., amount of the prodrug detected in the micelle fraction) over the incubation time of 24 h is shown in Figure 9b. The transfer of MlphDG from the ePC–MlphDG–PI liposomes to micelles was negligible with 1.0 and 1.3% prodrug detected in the micelle fraction in two independent experiments. Over the time course of incubation, the liposomes preserved their size according to MALS data (Supplementary Materials, Figure S7). Average recovery of MlphDG for all release measurements was 82.5 ± 8.3%.

Assessment of drug release from colloidal carriers, such as liposomes, is not trivial as the donor particles usually must be separated from the acceptor phase for quantification of released compound. The most frequently described method in the literature is dialysis which is, however, not applicable in most cases to study release of hydrophobic compounds from lipidic carriers [61]. Hydrophobic chlorins (e.g., mTHPC) and porphyrins (e.g., pTHPP, mTHPP) are not released to an aqueous acceptor phase due to their high partition coefficient (around 8–9) and insolubility in water [41], but addition of lipophilic acceptor particles results in a rapid release and redistribution to the acceptor [43]. Loew and colleagues [62] describe generally two drug transfer mechanisms, e.g., by diffusion through the water phase and by collision. For water insoluble compounds with considerably high partition coefficient, the collision mechanism is expected to be the main mechanism of drug redistribution. In the present study DSPE-mPEG micelles were used as acceptor phase at a 1:1 lipid mass ratio with the donor liposomes. Considering the size of around 100 nm, a single liposome contains ~80,150 lipid molecules. A DSPE-mPEG micelle with a size of around 15 nm contains some 225 molecules in brush regime with diameter of head group area ~20 Å [63]. Based on these considerations the micelle-to-liposome particle number ratio was roughly 100:1. The very slow release and redistribution of MlphDG observed in this study may be explained by the high partition coefficient of around 10 (Molinspiration service, https://www.molinspiration.com/services/logp.html) making its release by diffusion even over short distances unlikely. In addition, charge interactions between the positively charged MlphDG and the negatively charged PI may further contribute to the stable incorporation of the prodrug into the liposomal membrane. (Unfortunately, these interactions could not be distinguished in FTIR spectra among the signals of numerous bonds of other molecules forming the bilayer). The reliable retention of the MlphDG prodrug in the liposome bilayer, together with the colloidal stability of the ePC–MlphDG–PI formulation in human serum at least for 4 h [29], is advantageous in terms of delivery of the drug to target site in the body. As for the ultimate release of the drug, there are no data on the release of unmodified MlphDG from the liposomes in physiological milieu yet. Nevertheless, the results of antitumor activity and liposome localization studies in mouse models under systemic administration [27,28] evidence that the formulation performs its drug carrier function and the prodrug is available at the target site.

## 4. Conclusions

Our results show that the introduction of phosphatidylinositol in the fluid-phase liposomes loaded with a diglyceride ester of melphalan provides reliable protection of the bilayer against the insertion of serum albumin. Neither ester carbonyls of lipids nor fatty acyl chains respond to the interaction with the protein molecules (Figure 4, Table 4). Slight shift of the phosphate group bands (Table 3) evidences protein adsorption on the surface of liposomes. The lack of significant changes in the secondary structure of albumin also confirms the absence of strong interactions between the protein and the liposome membrane (Figure 7). On the contrary, incorporation of MlphDG alone in the ePC bilayer promotes penetration of albumin in the membrane, which is likely due to electrostatic attraction accompanied by the essential rearrangements in the protein structure. Interestingly, PI-containing liposomes without the melphalan prodrug were slightly more sensitive to the interactions with the protein than the ePC liposomes, as evidenced by some responses from phosphate groups and ester carbonyls (Figure 4, Table 4) and the decrease in α-helical structure content in albumin (Figure 7). Presumably, interactions between positively charged and small size melphalan moieties of the prodrug and negatively charged voluminous *mio*-inositol head groups of phosphatidylinositol make phosphates and carbonyls less accessible for interaction with albumin and thus prevent the protein invasion. Our findings obtained using asymmetrical flow field-flow fractionation technique also evidence high stability of the ePC–MlphDG–PI liposomes, particularly in the medium containing lipoprotein-mimicking particles, which can provoke collision-induced dissociation of the bilayer.

A scalable synthesis of MlphDG and a protocol for the production of lyophilized form of the ePC–MlphDG–PI liposomes has been developed that allowed us to fabricate a sufficient amount (liters) of the formulation and start preclinical trials [26]. The data on acute toxicity, tolerance and antitumor efficacy of the formulation in comparison with the parent drug melphalan are encouraging. Further progress implies studies of pharmacokinetics and bio-distribution of the liposomal prodrug and the released cytotoxic entity.

## Figures and Tables

**Table 1:** Asymmetrical flow field-flow fractionation (AF4) separation conditions applied in the release experiments. The injection and detector flow were set to 0.2 mL/min and 1 mL/min, respectively.

Mode	Duration, min	Cross/Focus Flow Start, mL/min	Cross/Focus Flow End, mL/min
Elution	1	1	1
Focus	1	2	2
Focus and inject	5	2	2
Focus	3	2	2
Elution	10	2	2
Elution	35	0	0
Elution and inject	5	0	0

**Table 2:** Composition and physicochemical characteristics of liposome samples as assessed by dynamic light scattering using the Malvern equipment.

Sample Composition, by mol	Z-Average Size, nm ^1^	PdI Width, nm (Average)	PdI	Zeta-Potential, mV ^2^
ePC	121.3 ± 2.4	35.2 ± 1.1	0.084 ± 0.003	−1.4 ± 1.6
ePC–MlphDG, 9:1	117.3 ± 2.2	33.1 ± 1.1	0.079 ± 0.003	+18.4 ± 1.9
ePC–PI, 9:1	104.4 ± 1.8	28.5 ± 2.6	0.068 ± 0.016	−48.8 ± 2.6
ePC–MlphDG–PI, 8:1:1	121.3 ± 2.3	34.8 ± 1.1	0.082 ± 0.003	−25.3 ± 1.9

^1^ Mean values ± SD are presented. Size distribution curves can be found in Supplementary Materials, Figure S1. ^2^ Data for 200-nm liposomes were obtained using ZetaPALS analyzer (Brookhaven Instruments Corp.).

**Table 3:** Changes in frequencies (cm^−1^) of asymmetric stretching vibrations of phosphate groups upon incubations of different liposome samples with serum albumin.

Liposome Composition	Before Incubation	5-Min Incubation
ePC	1230.4	1230.9
ePC–MlphDG, 9:1	1231.3	1238.6
ePC–PI, 9:1	1229.9	1233.3
ePC–MlphDG–PI, 8:1:1	1228.4	1230.9

**Table 4:** Stretching vibration frequencies (cm^−1^) of lipid methylene groups upon incubation of different liposome samples with serum albumin.

Liposome Composition	Before Incubation		5-Min Incubation	
	ν_as_	ν_sim_	ν_as_	ν_sim_
ePC	2923.6	2853.2	2923.6	2853.2
ePC–MlphDG, 9:1	2923.7	2853.2	2925.5	2853.7
ePC–PI, 9:1	2924.1	2853.2	2924.5	2853.2
ePC–MlphDG–PI, 8:1:1	2924.1	2853.2	2924.1	2853.2

**Table 5:** Correlation between BSA secondary structure and amide I second derivative position ^1^.

Secondary Structure	Range, cm^−1^
α-helix	1650–1660
β-sheet	1628–1639
β-turn	1664–1687
random coil	1640–1649
Intermolecular β-sheet (aggregates)	1618–1626, 1688–1696

^1^ Usually, only beta-structures are assigned to multiple bands (e.g., see [54]); expanded ranges for α-helix and random coil peak positions were used as proposed in [55] for albumin.

**Table 6:** Size values by dynamic light scattering (DLS, *n* = 10) and AF4/multi-angle light scattering (MALS, *n* = 3).

Sample	DLS		AF4			
	D_h_, nm	PdI	D_10_, nm	D_50_, nm	D_90_, nm	D_mean_, nm
ePC–PI, 9:1	104 ± 2	0.091 ± 0.025	78.6 *	104.4 *	120.6 *	106.4 *
ePC–MlphDG–PI, 8:1:1	95 ± 2	0.132 ± 0.011	75.2 ± 1.3	100.5 ± 0.3	120.7 ± 0.5	104.1 ± 0.5
DSPE-PEG (micelles)	15 ± 1	0.310 ± 0.148	n.a.			

* Single analysis.

## Supplementary Materials

The following are available online at https://www.mdpi.com/article/10.3390/pharmaceutics13040473/s1, Figure S1: comparison of size distribution curves obtained for different liposome samples with Zetasizer Nano ZS (Malvern Panalytical Ltd., UK) (a). Example of size distribution curves obtained for one sample (ePC–PI) during 3 measurement runs (b), Figure S2: excitation and emission spectra of FITC (green) and BCHB-PC (orange) (a). BSA-titration graph (b). FITC signal decrease after liposome addition with a different BSA to liposomes molecule ratio, Figure S3: PO_2_^−^ peaks for ePC (a), ePC–MlphDG (b), ePC–PI (c), ePC–MlphDG–PI (d) liposomes alone and upon their incubation with BSA, Figure S4: ester carbonyl peaks for ePC (a), ePC–MlphDG (b), ePC–PI (c), ePC–MlphDG–PI (d) liposomes alone and upon their incubation with BSA, Figure S5: methylene peaks for the ePC–PI (a) and ePC–MlphDG–PI (b) liposomes alone and upon their incubation with BSA, Figure S6: Amide I peaks for pure BSA (blue dashed line) and BSA incubated with the ePC (a), ePC–MlphDG (b), ePC–PI (c), ePC–MlphDG–PI (d) liposomes, Figure S7: AF4/MALS elution profile of micelles and ePC–MlphDG–PI liposomes injected in mixture during 24 h incubation.

## Abbreviations

AF4, asymmetrical flow field-flow fractionation; ATR-FTIR, attenuated total reflection Fourier transform infrared spectroscopy; BSA, bovine serum albumin; BCHB-PC, bis-cyclohexyl-BODIPY-labeled phosphatidylcholine; DMPC, 1,2-dipalmitoyl-sn-glycero-3-phosphocholine; DOPC, 1,2-dioleoyl-sn-glycero-3-phosphocholine; POPC, 1-palmitoyl-2-oleoyl-sn-glycero-3-phosphocholine; DOTAP, 1,2-dioleoyl-3-trimethylammonium propane; DPPC, 1,2-dipalmitoyl-sn-glycero-3-phosphocholine; DSPE-mPEG, 1,2-distearoyl-sn-glycero-3-phosphoethanolamine with conjugated methoxyl poly(ethylene glycol)-2000; ePC, egg yolk phosphatidylcholine; MALS, multi-angle light scattering; MlphDG, melphalan ester conjugate with 1,2-dioleoyl-sn-glycerol; PI, phosphatidylinositol; VWD, variable wavelength detector.
