# Supplementary material for: Phosphatidylinositol Stabilizes Fluid-Phase Liposomes Loaded with a Melphalan Lipophilic Prodrug
Source: Pharmaceutics. 2021 Apr 1;13(4):473. doi: 10.3390/pharmaceutics13040473 (PMC8067299; doi:10.3390/pharmaceutics13040473)
Supplement: Supplementary file 1 [file pharmaceutics-13-00473-s001.pdf]

pharmaceutics

Supplementary Materials: Phosphatidylinositol Stabilizes Fluid-Phase Liposomes Loaded with a Melphalan Lipophilic Prodrug

Daria Tretiakova, Irina Le-Deigen, Natalia Onishchenko, Judith Kuntsche, Elena Kudryashova and Elena Vodovozova*

1. Liposome characterization

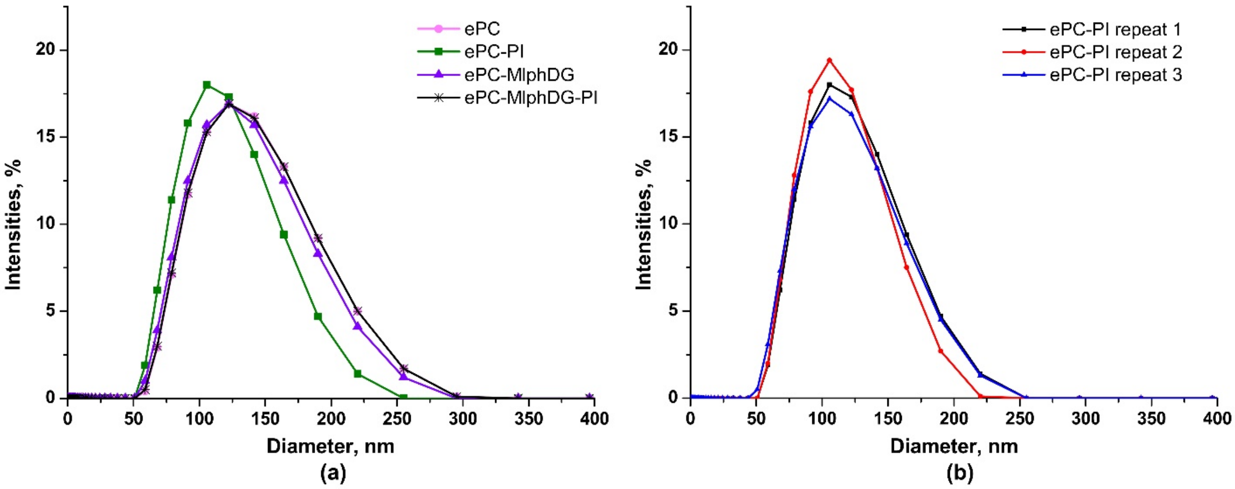

Figure S1. Comparison of size distribution curves obtained for different liposome samples with Zetasizer Nano ZS (Malvern Panalytical Ltd., UK) (**a**). Example of size distribution curves obtained for one sample (ePC–PI) during 3 measurement runs (**b**).

2. Förster resonance energy transfer using FITC-BSA conjugate as a donor and BCHB-PC probe in liposome bilayer as an acceptor molecule.

For the **FITC-BSA** conjugate, we calculated the dye/protein (D/P) ratio. D/P value of several batches of FITC-BSA conjugate had small variation from 1.5 to 1.6.

For FITC and bis-cyclohexyl-BODIPY-labeled phosphatidylcholine (BCHB-PC) [1] probes, we calculated Förster radius to be 57.6 Å. When added to liposomes, BSA conjugate signal decreased due to Förster resonance energy transfer and stayed constant for 20 min of incubation.

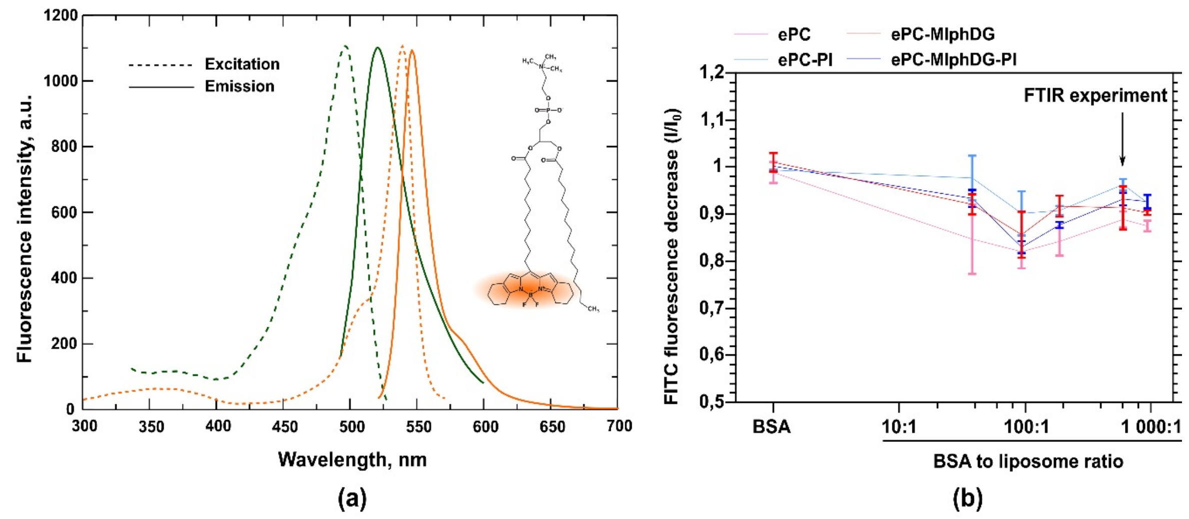

Figure S2. Excitation and emission spectra of FITC (green) and BCHB-PC (orange) (**a**). BSA-titration graph (**b**). FITC signal decrease after liposome addition with a different BSA molecules-to-liposomes ratio.

3. ATR-FTIR Spectroscopy. Asymmetric stretching vibrations of lipid phosphate groups.

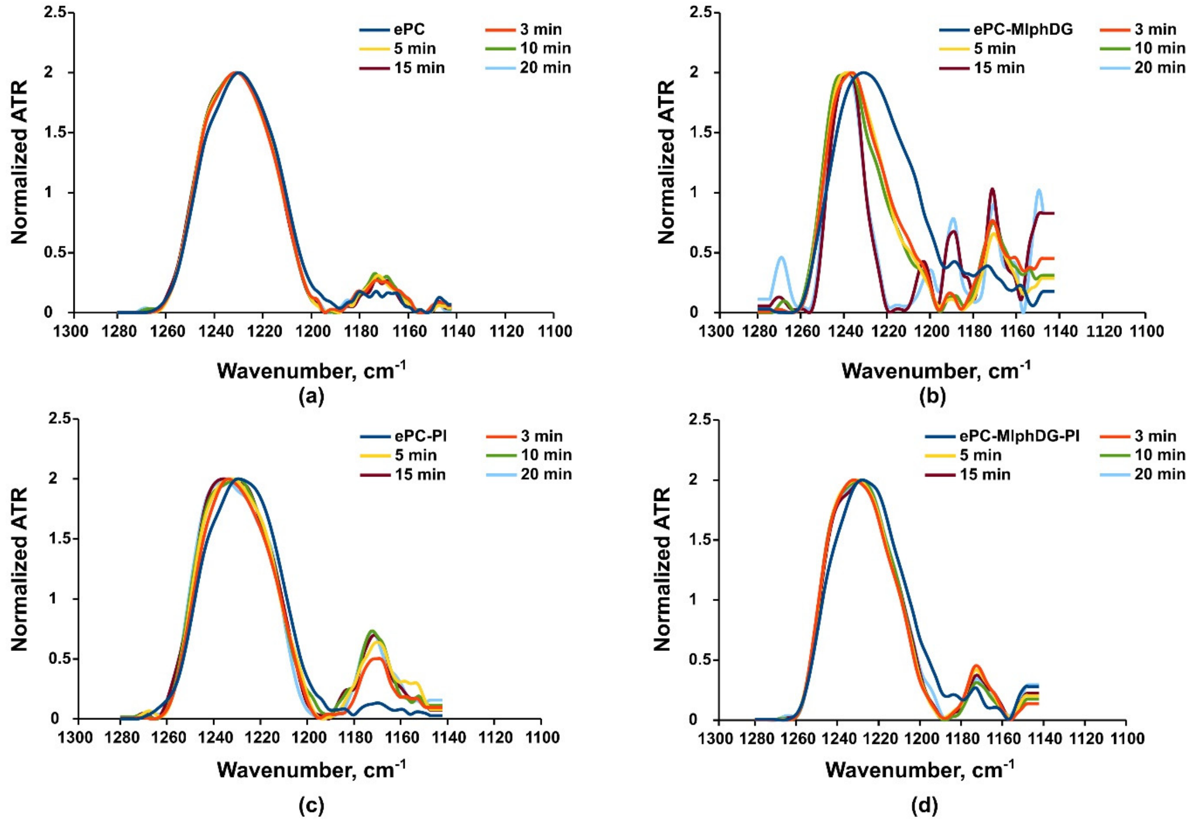

Figure S3. PO_2^- peaks for ePC (**a**), ePC–MlphDG (**b**), ePC–PI (**c**), ePC–MlphDG–PI (**d**) liposomes alone and upon their incubation with BSA.

Lipid ester carbonyl stretching bands.

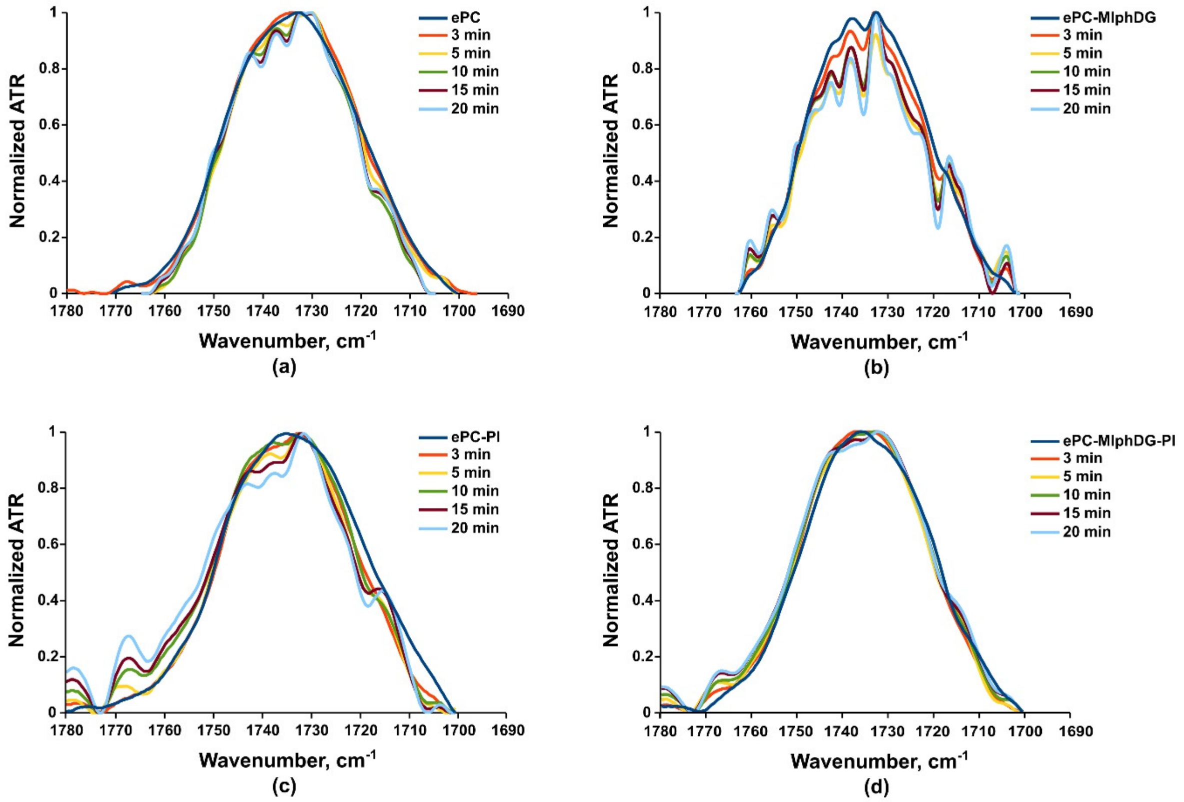

Figure S4. Ester carbonyl peaks for ePC (**a**), ePC–MlphDG (**b**), ePC–PI (**c**), ePC–MlphDG–PI (**d**) liposomes alone and upon their incubation with BSA.

Lipid methylene stretching vibrations.

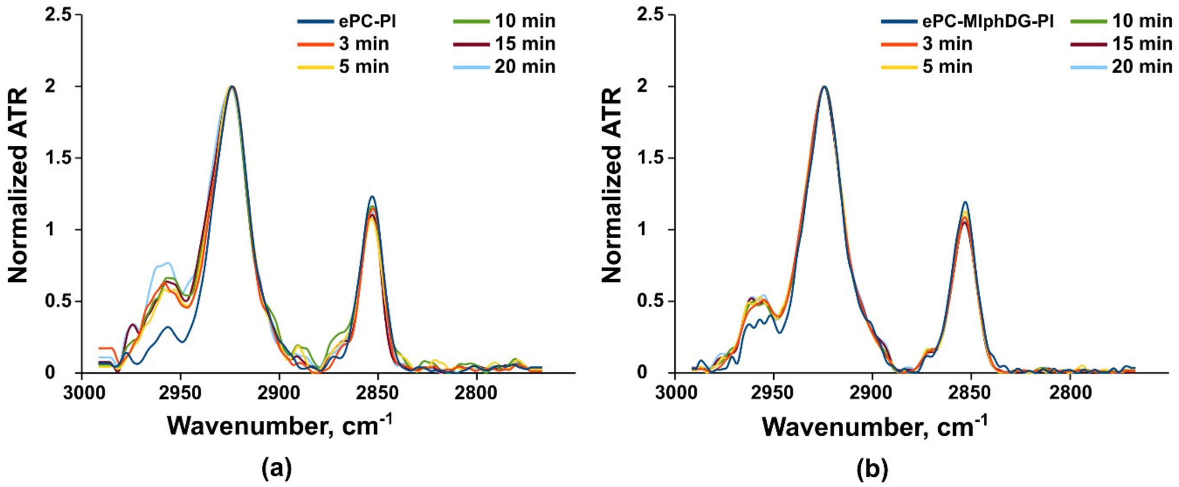

Figure S5. Methylene peaks for the ePC–PI (**a**) and ePC–MlphDG–PI (**b**) liposomes alone and upon their incubation with BSA.

Protein amide I mode.

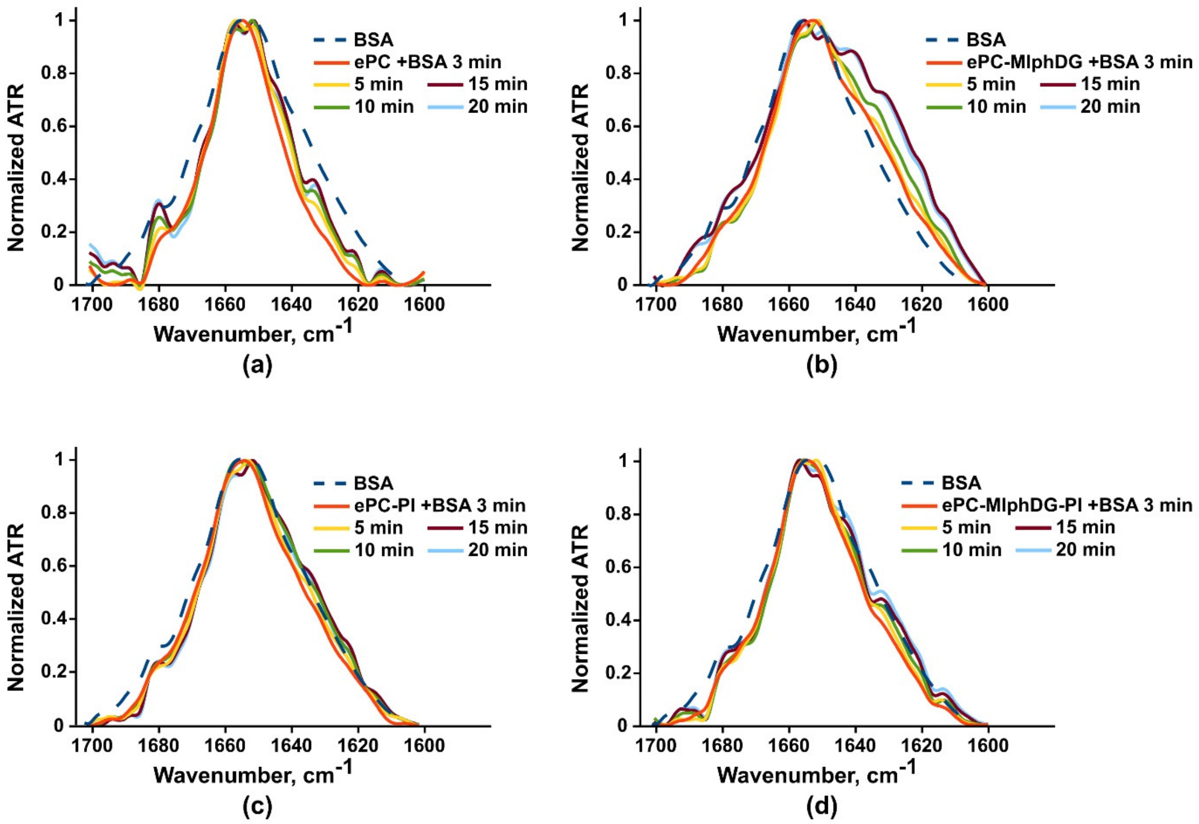

Figure S6. Amide I peaks for pure BSA (blue dashed line) and BSA incubated with the ePC (**a**), ePC-MlphDG (**b**), ePC-PI (**c**), ePC-MlphDG-PI (**d**) liposomes.

4. Asymmetrical Flow-Field-Flow Fractionation (AF4). Liposome size stability.

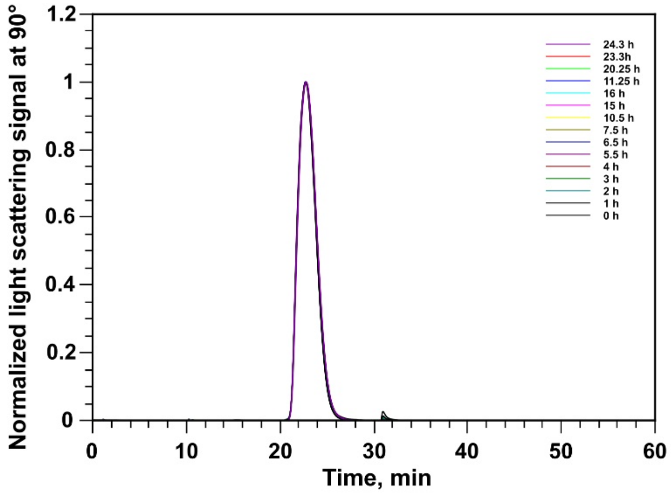

Figure S7. AF4/MALS elution profile of micelles and ePC–MlphDG–PI liposomes injected in mixture during 24h incubation.

References

1. Tretiakova, D. S., Alekseeva, A. S., Galimzyanov, T. R., Boldyrev, A. M., Chernyadyev, A. Y., Ermakov, Y. A., Batishchev, O. V., Vodovozova, E. L., and Boldyrev, I. A. (2018) Lateral stress profile and fluorescent lipid probes. FRET pair of probes that introduces minimal distortions into lipid packing. Biochim. Biophys. Acta, Biomembr. 1860, 2337–2347.
